# A Review of Recent Developments in Biopolymer Nano-Based Drug Delivery Systems with Antioxidative Properties: Insights into the Last Five Years

**DOI:** 10.3390/pharmaceutics16050670

**Published:** 2024-05-16

**Authors:** Magdalena Stevanović, Nenad Filipović

**Affiliations:** Group for Biomedical Engineering and Nanobiotechnology, Institute of Technical Sciences of SASA, Kneza Mihaila 35/IV, 11000 Belgrade, Serbia; nenad.filipovic@itn.sanu.ac.rs

**Keywords:** biopolymers, nanoparticles, antioxidants, polysaccharides, polynucleotides, proteins polyesters

## Abstract

In recent years, biopolymer-based nano-drug delivery systems with antioxidative properties have gained significant attention in the field of pharmaceutical research. These systems offer promising strategies for targeted and controlled drug delivery while also providing antioxidant effects that can mitigate oxidative stress-related diseases. Generally, the healthcare landscape is constantly evolving, necessitating the continual development of innovative therapeutic approaches and drug delivery systems (DDSs). DDSs play a pivotal role in enhancing treatment efficacy, minimizing adverse effects, and optimizing patient compliance. Among these, nanotechnology-driven delivery approaches have garnered significant attention due to their unique properties, such as improved solubility, controlled release, and targeted delivery. Nanomaterials, including nanoparticles, nanocapsules, nanotubes, etc., offer versatile platforms for drug delivery and tissue engineering applications. Additionally, biopolymer-based DDSs hold immense promise, leveraging natural or synthetic biopolymers to encapsulate drugs and enable targeted and controlled release. These systems offer numerous advantages, including biocompatibility, biodegradability, and low immunogenicity. The utilization of polysaccharides, polynucleotides, proteins, and polyesters as biopolymer matrices further enhances the versatility and applicability of DDSs. Moreover, substances with antioxidative properties have emerged as key players in combating oxidative stress-related diseases, offering protection against cellular damage and chronic illnesses. The development of biopolymer-based nanoformulations with antioxidative properties represents a burgeoning research area, with a substantial increase in publications in recent years. This review provides a comprehensive overview of the recent developments within this area over the past five years. It discusses various biopolymer materials, fabrication techniques, stabilizers, factors influencing degradation, and drug release. Additionally, it highlights emerging trends, challenges, and prospects in this rapidly evolving field.

## 1. Introduction

The continuous struggle in healthcare demands improved therapeutic approaches, which also involves the development of new drugs or systems for their delivery [1,2,3]. The challenges and expectations in this field bring versatile research approaches. One of them that imposes great potential is certainly in creating drug delivery systems (DDSs) [4,5]. A DDS refers to a formulation or device enabling the administration of a therapeutic substance into the body [6]. Advancements in DDSs have revolutionized the field of medicine, allowing for more effective treatments, reduced dosing frequency, improved patient compliance, and minimized adverse effects [7,8]. Tailoring DDSs to specific drugs and therapeutic needs continues to be an active area of research and development in the pharmaceutical industry [9]. DDSs are designed to release a drug at a predetermined rate, location, and time interval to optimize therapeutic outcomes while minimizing side effects [10]. Until now, numerous drug delivery systems have been developed to optimize the pharmacokinetics and pharmacodynamics of medicaments [11]. They encompass oral administration, injectables, transdermal patches, inhalation methods, implantable delivery systems, targeted drug delivery, and nanotechnology-driven delivery approaches [5,12]. While each of these systems has its merits, the focus here will be on nanotechnology-driven delivery approaches.

Nanomaterials offer unique properties for drug delivery, such as improved solubility, controlled release, and targeted delivery to specific cells or tissues [13,14,15,16,17]. Generally, nanotechnology involves the study, manipulation, and application of materials and structures at the nanoscale level [18,19]. This field encompasses various disciplines, including physics, chemistry, biology, engineering, and materials science, and has led to the development of diverse nanomaterials with distinctive characteristics and applications across multiple industries [7,20]. Some common types of nanomaterials include nanoparticles, nanocapsules, nanospheres, nanotubes, quantum dots, nanocomposites, etc. [21]. As an illustration, nanoparticles possess dimensions within the nanoscale realm, providing a notable surface area-to-volume ratio [22]. These nanoparticles, crafted from diverse materials like metals, metal oxides, polymers, or carbon-based substances, serve various purposes in drug delivery, tissue engineering, and imaging [23,24]. Similarly, nanotubes, comprising cylindrical arrangements of carbon atoms, are prized for their exceptional mechanical, electrical, and thermal attributes, making them popular in biomedical domains [25]. Quantum dots, semiconductor nanocrystals, demonstrate distinctive quantum mechanical traits, including size-adjustable light emission [26]. They are used in displays, biological imaging, and photovoltaic devices. Having a particularly important role in the fields of medicine and pharmacy, nanocomposites are materials that consist of a combination of nanoparticles or nanoscale additives dispersed within a bulk material matrix [27]. Applications of nanotechnology and nanomaterials span a wide range of fields, including medicine for drug delivery, diagnostics, tissue engineering, etc. However, the stability, release profile, and overall bioavailability are still the main requirements that need to be accomplished at a satisfying level when developing new or enhancing already existing DDSs.

**Figure 1:**
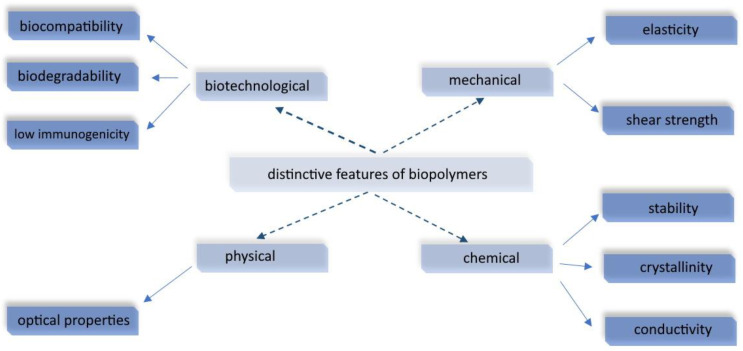
Distinctive features of biopolymers.

Biopolymer-based drug delivery systems offer a promising approach for the targeted and controlled release of therapeutic agents [28,29,30]. These systems utilize natural or synthetic biopolymers to encapsulate drugs, protect them from degradation, and deliver them to specific sites within the body [31,32,33]. The use of biopolymers in drug delivery systems offers several advantages, including biocompatibility, biodegradability, low immunogenicity, and the ability to tailor their properties for specific applications. There is a long list of distinctive features of biopolymers, as shown in Figure 1. Biopolymers are large molecules composed of repeating structural units (monomers) linked together through covalent bonds. Because of all the properties mentioned above, they are often used in this area [34]. They have a wide range of applications in different industries such as medicine, food science, agriculture, and biotechnology. For instance, biodegradable polymers derived from natural sources are used in sustainable packaging materials, tissue engineering, drug delivery systems, and bio-based materials production [35,36].

Biopolymer-based drug delivery systems can be used to control the release of drugs over an extended period, which can improve patient compliance and reduce the frequency of drug administration [37]. For these purposes, the mainly used biopolymers are polysaccharides (chitosan, alginate, cellulose, starch, hyaluronic acid, dextran), polynucleotides, polyesters (polylactide, poly(lactide-co-glycolide), polycaprolactone, etc.), and proteins (silk fibroin, collagen, gelatine, and albumin) [33,38,39,40,41,42,43]. Proteins are biopolymers made up of amino acid monomers linked by peptide bonds [44]. They serve various functions in the body, including structural support, enzyme catalysis, immune response, and cell signaling. Polysaccharides are complex carbohydrates made of monosaccharide units [45]. Polyesters are a class of biodegradable polymers obtained from biological monomers like lactic acid, glycolic acid, hydroxybutyrate, and caprolactone [46,47]. Nucleic acid biopolymers are composed of nucleotide monomers. They store and transmit genetic information in cells [48]. All these biopolymers hold the potential for creating nano-based drug delivery systems that exhibit antioxidative effects and have been the subject of extensive research efforts.

Substances or compounds demonstrating antioxidative properties play a crucial role in reducing the risk of various diseases, as evidenced in many articles [49,50,51,52,53,54].

**Figure 2:**
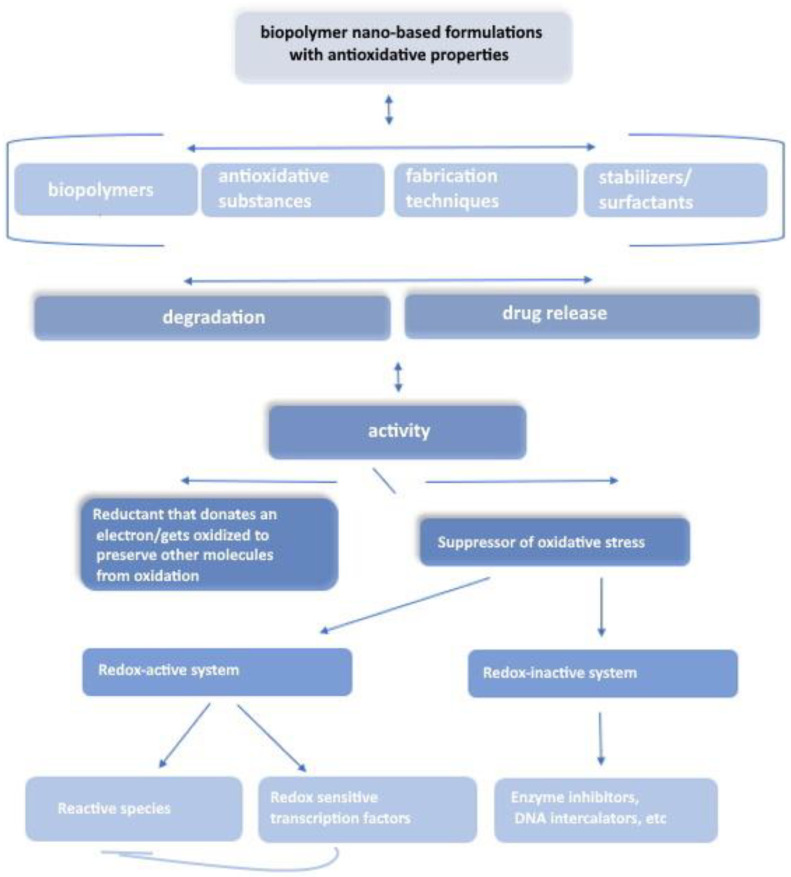
Scheme representing the purpose behind biopolymer nano-based drug delivery systems with antioxidative properties.

These compounds serve to potentially prevent or mitigate damage caused by oxidation, which occurs due to free radicals—unstable molecules containing unpaired electrons [55]. Free radicals tend to react with other molecules in the body, inducing oxidative damage to cells, proteins, lipids, and DNA. This oxidative stress is closely associated with a spectrum of diseases, aging processes, and tissue damage. Antioxidants function as compounds that inhibit or counteract the detrimental effects of oxidative stress by providing electrons to neutralize free radicals, thereby stabilizing them [56,57,58]. Sources rich in antioxidants encompass a variety of options, including fruits, vegetables, nuts, seeds, herbs, and select beverages [53,59]. Common antioxidants include vitamins, polyphenols, flavonoids, and carotenoids. The roles of antioxidants are extensive, spanning from neutralizing free radicals and averting cellular and tissue oxidative damage to shielding against chronic diseases like heart disease, cancer, and neurodegenerative disorders. Moreover, antioxidants support the body’s defense mechanisms against environmental stressors and toxins, contributing to the maintenance and enhancement of overall health and well-being. Numerous research endeavors focus on developing systems featuring antioxidant properties, aiming to amplify the antioxidant effect and extend its duration (Figure 2) [60].

Since 2019, approximately 16,400 research and review articles related to biopolymer antioxidative drug delivery systems have been published according to Google Scholar. Interestingly, the number of articles has increased year by year, which shows the huge research interest in this area. This is also supported by the number of articles according to the index base Scopus searched by keywords “biopolymer + antioxidative + drug + delivery + systems”, which amounts to 2449 documents for the period 2019–2024, with a peak in 2023 which unequivocally indicates the actuality of this research field.

Hence, it is essential to organize existing knowledge systematically to facilitate continued advancements in research. This review aims to elucidate the present status of biopolymer-based nanoformulations functioning as drug delivery systems with antioxidative properties. It encompasses various aspects such as synthesis methodologies, commonly utilized incorporation techniques, surface modification using stabilizers, surface functionalization, factors influencing degradation, drug release kinetics, and more. These aspects are explored within the context of nanoformulations, categorized in this review according to the biopolymer types, namely (i) polysaccharide-based nanoformulations, (ii) polynucleotide-based nanoformulations, (iii) protein-based nanoformulations, and (iv) polyester-based nanoformulations. Table 1 shows the main biopolymer types and their chemical structures.

The selection and insertion of papers in this review followed a structured process to ensure the inclusion of the relevant and high-quality literature. This process has been conducted based on a comprehensive literature search using the academic databases Scopus, PubMed, Web of Science, and Google Scholar. A combination of keywords related to biopolymer-based drug delivery systems, antioxidants, nanotechnology, and drug delivery have been used in this process. After compiling a list of relevant articles, the titles and abstracts were screened to identify papers that met the inclusion criteria. These criteria have included relevance to the topic, recent publication date, and focus on biopolymer-based nano-drug delivery systems with antioxidative properties. The selected papers then underwent a full-text review to assess their suitability for inclusion in this review article based on the evaluated methodology, results, and discussion of each paper.

Ultimately, this review will comprehensively evaluate both the advantages and limitations associated with complex biopolymer nano-based delivery systems. It will delve into the intricacies of these systems, highlighting their potential benefits such as enhanced drug stability, targeted delivery, and controlled release kinetics. Additionally, the review will address the challenges and drawbacks inherent in the design and fabrication, including techniques such as ionic gelation, nanoprecipitation, crosslinking, self-assembly, coacervation, or emulsion-based techniques. Moreover, the review will shed light on the current research landscape within this domain, emphasizing emerging trends, innovative methodologies, and promising avenues for future exploration. By synthesizing the latest findings and identifying key research focal points, the review aims to provide valuable insights to researchers, practitioners, and stakeholders in the field of drug delivery.

## 2. Polysaccharide-Based Nanoformulations

### 2.1. Polysaccharides

Polysaccharides are complex carbohydrates composed of long chains of monosaccharide units linked together by glycosidic bonds. Chitosan, alginate, cellulose, starch, hyaluronic acid, and dextran are examples of polysaccharides with diverse properties and applications [61]. Chitosan is a biopolymer derived from chitin, which is found in the shells of crustaceans. Chitosan-based formulations have been used for the delivery of a variety of drugs, including antibiotics, anticancer agents, and growth factors [62,63]. Chitosan can be formulated into nanoparticles, microspheres, or hydrogels, which can control the release of drugs over a period of hours to weeks [64]. In the last few years, it has been widely used to obtain nanoparticles that have antioxidant properties [65,66,67,68,69,70,71,72,73,74,75,76,77,78]. Alginate is a natural polysaccharide that is derived from seaweed. Alginate-based formulations have often been used for the delivery of proteins, peptides, and small molecules [79]. Alginate hydrogels find applications in drug delivery systems, cell encapsulation for tissue engineering, and wound dressings due to their biocompatibility. Cellulose is the most abundant natural polymer and a major component of plant cell walls. It possesses excellent mechanical strength, biocompatibility, and biodegradability. Cellulose and its derivatives are often used in industries for manufacturing pharmaceuticals and as a biomaterial in tissue engineering and wound healing applications [80,81,82,83,84,85,86,87]. Starch is a carbohydrate found in various plant-based foods, such as grains, tubers, and legumes. It serves as an energy reserve in plants. Starch finds applications in biodegradable plastics, adhesives, and pharmaceuticals [88,89]. Hyaluronic acid (HA), also known as hyaluronan, is a naturally occurring linear polysaccharide composed of repeating units of glucuronic acid and N-acetylglucosamine. It is found abundantly in connective tissues, skin, eyes, and synovial fluid in joints [90]. HA plays a role in tissue regeneration and wound healing by promoting cell migration and proliferation. It contributes to the repair process and has been used in various medical applications to aid in wound closure and tissue regeneration. Due to its biocompatibility, biodegradability, and various beneficial properties, hyaluronic acid has found widespread applications in medicine, skincare, ophthalmology, orthopedics, and tissue engineering. Ongoing research continues to explore its potential applications and optimize its use in diverse fields [91,92,93]. Dextran is produced by certain strains of bacteria, particularly Leuconostoc mesenteroides and Streptococcus mutans, through the fermentation of sucrose or other sugars. Key characteristics of dextran include its water-solubility, biocompatibility, and ability to form gels [94]. Dextran molecules vary in size, and their molecular weights can range from relatively small to very large, depending on the production process and intended use [95]. Dextran’s versatility and properties make it a valuable compound with applications in various fields, particularly in medicine, biotechnology, and the food industry [96].

**Figure 3:**
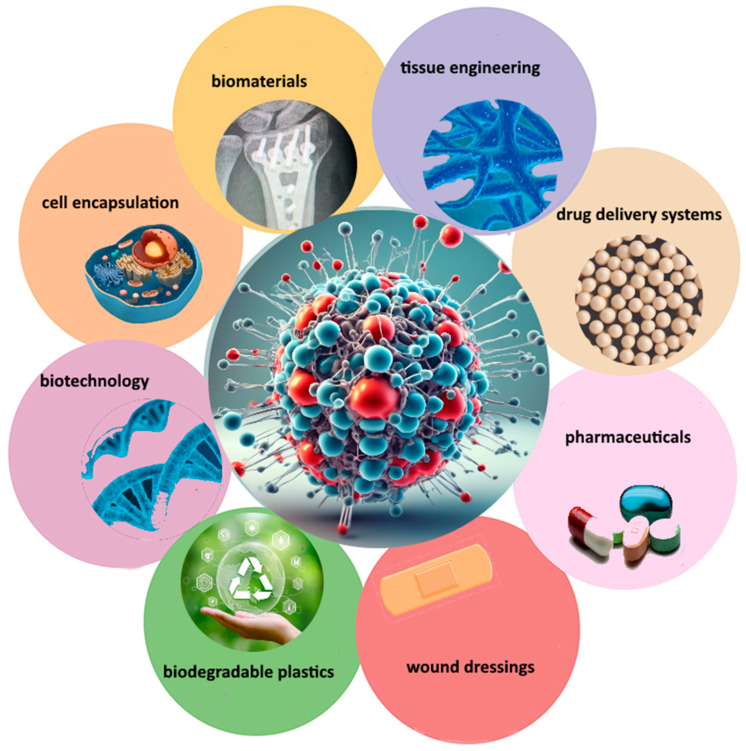
Different applications of polysaccharides.

Ongoing research aims to further explore its potential uses and optimize its functionalities for different applications (Figure 3). All these polysaccharides exhibit unique properties that make them valuable in diverse industries, including healthcare and materials science.

### 2.2. Different Formulations

**Figure 4:**
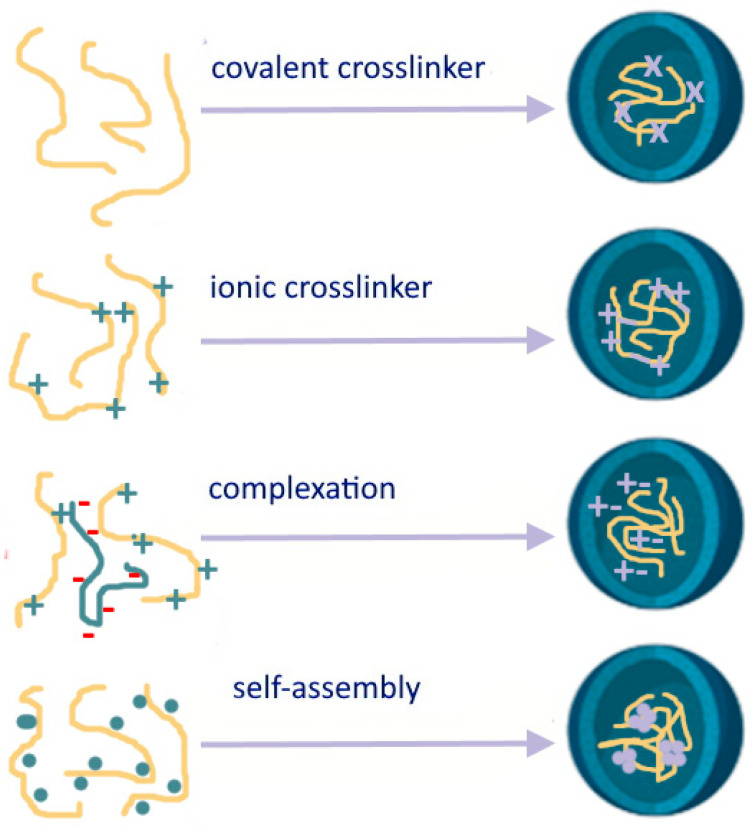
Scheme illustrating mechanisms generally involved in the synthesis of polysaccharide nanoparticles.

Polysaccharide nanoformulations have gained significant attention in drug delivery due to their biocompatibility, biodegradability, and versatility. These nanoformulations involve incorporating drugs or therapeutic agents into polysaccharide-based nanocarriers to enhance drug stability, solubility, and targeted delivery to specific tissues or cells. Various polysaccharides, already mentioned above, such as chitosan, alginate, hyaluronic acid, cellulose, dextran, and others, have been utilized in the development of nanoformulations for drug delivery purposes and with antioxidative properties [97,98,99,100,101]. In their detailed review, Mizrahy and Peer thoroughly examined the primary mechanisms generally involved in the synthesis of polysaccharide nanoparticles [102]. These mechanisms include chemical (covalent) crosslinking, physical (ionic) crosslinking, polyelectrolyte-complexation, and self-assembly (Figure 4).

#### 2.2.1. Nanoparticles

Polysaccharides can be formulated into nanoparticles through the most commonly used techniques, such as ionic gelation, nanoprecipitation, crosslinking, self-assembly, coacervation, or emulsion-based techniques. These nanoparticles can encapsulate drugs, protecting them from degradation and enabling controlled release.

**Figure 5:**
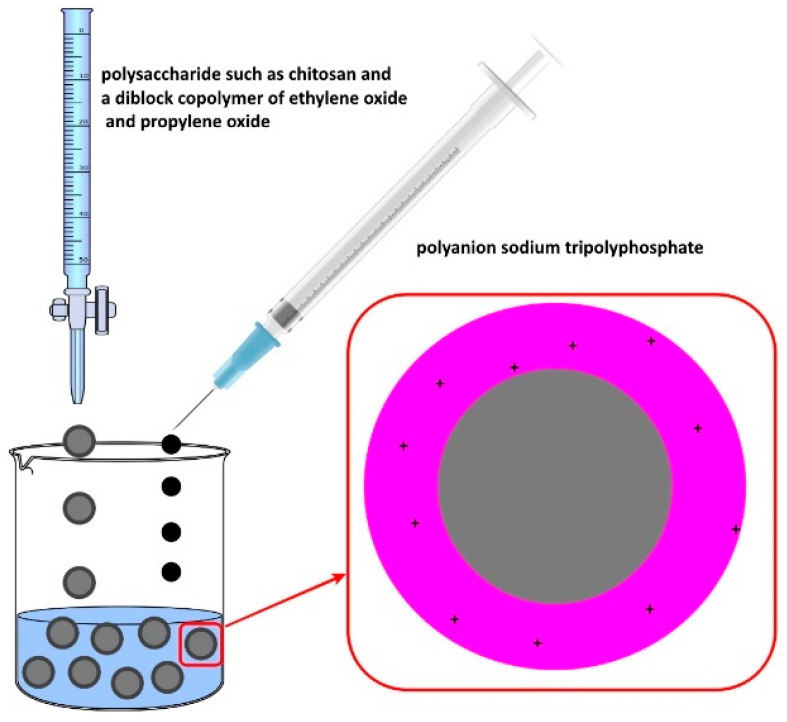
Illustration of the ionic gelation technique.

There are many examples of research performed to obtain chitosan nanoparticles with an antioxidant effect. One of them is recent work conducted by Hanna and coworkers in which chitosan nanoparticles containing naringenin were synthesized by an ionic gelation method mediated by tripolyphosphate as a crosslinker [103]. **The ionic gelation technique**, discovered by Calvo et al., is a chemical approach utilized for synthesizing microparticles or nanoparticles [104,105]. It relies on electrostatic interactions between ions of opposite charges and typically involves the use of polymers, such as chitosan and alginate [106]. The ionic gelation process is exceptionally mild, entailing the combination of two aqueous phases at ambient temperature. One phase usually contains the polysaccharide, such as chitosan, and a diblock copolymer of ethylene oxide and propylene oxide, and the other contains the polyanion sodium tripolyphosphate (Figure 5).

The chosen chitosan nanoparticle formulation, produced in the study mentioned above of Hanna et al., exhibited a 5% drug loading and a size of 150 nm and possessed cationic properties. Intranasal administration of naringin improved memory function, suppressed hippocampal acetylcholinesterase activity, and mitigated oxaliplatin-induced histological alterations. Additionally, it decreased hippocampal malondialdehyde levels while increasing reduced glutathione levels. Moreover, it attenuated inflammatory marker levels and downregulated protein levels in two pathways upstream of hippocampal inflammation [103]. By the same method, ionic gelation, chitosan-tripolyphosphate nanoparticles were prepared by Kim and coworkers to improve the antioxidant activities of astaxanthin. The findings indicate that encapsulating astaxanthin within chitosan-tripolyphosphate nanoparticles effectively boosts its stability, antioxidant characteristics, and bioavailability [107]. The ionic gelation method was used recently for the synthesis of chitosan nanoparticles with antioxidative properties by different research groups. The study of Jardim et al. aimed to nanoencapsulate quercetin within chitosan nanoparticles cross-linked using ionic gelation with sodium tripolyphosphate [108]. The goal was to investigate the release behavior of quercetin and assess its cytotoxicity and antioxidant activity in vitro using tumor cells and the DPPH method, respectively. The authors observed that chitosan/tripolyphosphate/quercetin nanoparticles showed a greater antioxidant effect when compared to free quercetin. The objective of the research conducted by Canbolat et al. was to fabricate novel nanoparticles by incorporating quercetin (Que) and valproic acid (VPA) into chitosan produced by the ionic gelation method [109]. The study investigated the antioxidant properties of chitosan NPs loaded with single and combined drugs (Que and VPA) against oxidative stress. The methodology encompassed the synthesis of chitosan NPs loaded with Que, VPA, and their combination; characterization of the NPs; in vitro antioxidant activity assessments; and the evaluation of cytotoxicity and antioxidant efficacy using human neuroblastoma SH-SY5Y cell lines. The authors inferred that the antioxidant effect of chitosan likely stemmed from its capacity to mitigate intracellular oxidative stress mechanisms rather than directly inhibiting free radicals, as evidenced by cellular oxidative stress analyses. Nanoparticles with quercetin were also developed by Ma et al. [110]. They developed zein/chitosan nanoparticles for encapsulating quercetin to overcome its lower water solubility and instability and to concomitantly enhance its cellular uptake and intracellular antioxidant activity.

Anthocyanins, flavonoids with various health benefits, face challenges due to low chemical stability and bioavailability. Nanoencapsulation with biopolymers has emerged as a promising approach to stabilize them. The study of Chatterjee et al. focuses on developing, characterizing, and assessing the antioxidant activity of chitosan nanoparticles obtained by ionic gelation and loaded with black carrot anthocyanin [111]. Lycopene, a member of the carotenoid family, exhibits favorable pharmacological properties such as antioxidant, anti-inflammatory, and anticancer effects. There are studies dealing with the investigation of the in vitro antioxidant activity of chitosan nanoparticles and their incorporation of lycopene [112,113].

This method was also recently used for obtaining alginate nanoparticles with the aqueous extract of C. grandis (AqCG) by de Silva et al. [114]. The primary objective of this study is to assess the antioxidant potential of alginate nanoparticles containing the aqueous extract of Coccinia grandis L. (Family: Cucurbitaceae). The aqueous extract of C. grandis (AqCG) was obtained through ultrasonication followed by refluxing. Alginate nanoparticles encapsulating the AqCG were synthesized using ionic gelation with the incorporation of extracts and CaCl2. The antioxidant efficacy of the nanoparticles was determined through in vitro assays, including the ferric reducing antioxidant (FRAP) assay, 2,2-diphenyl-1-picrylhydrazyl (DPPH) radical scavenging assay, and 2,2′-azino-bis(3-ethylbenzothiazoline-6-sulfonic acid) (ABTS) radical scavenging assay. Encapsulation notably enhanced the antioxidant activity, particularly observed in the ABTS assay compared to the AqCG alone.

The **nanoprecipitation method** has also been utilized very often to prepare various types of polysaccharide nanoparticles with antioxidative properties, offering versatility and rapidity in synthesis. For instance, starch nanoparticles were synthesized by adding dissolved starch solution into excess ethanol, with surfactants modulating their size and shape [115]. The choice of an appropriate synthesis method relies on the specific application and its corresponding criteria. Several considerations, including the thermal and chemical stability of the active ingredient, reproducibility of release kinetics, particle size, stability of the end product, and potential residual toxicity, must be considered before the formulation of drug delivery nanocarriers and nanoparticles for various applications [116].

Nanoprecipitation, also known as solvent displacement or interfacial deposition, is a well-established technique for encapsulating drug molecules, widely employed in the formation of various nanoparticles (NPs), including polymeric, drug-loaded proteins and inorganic NPs [117]. While offering simplicity, reproducibility, and cost-effectiveness, nanoprecipitation encounters challenges in encapsulating water-soluble compounds. In this process, hydrophobic solutes initially dissolved in a water-miscible solvent are added to an excess of an anti-solvent, resulting in the precipitation of hydrophobic compounds. The mixing of phases significantly influences particle size control, with good mixing conditions yielding smaller NPs. Additionally, surface tension disparity between the aqueous and organic phases plays a pivotal role in NP formation, where continuous solvent vortice formation leads to gradual precipitation of the polymers on the organic surface. Key parameters such as mixing temperature, solvent/antisolvent ratios, and polymer properties are crucial for controlling NP size and morphology in traditional, flash, and micro-fluidic nanoprecipitation methods. While traditional nanoprecipitation offers simplicity and affordability, flash nanoprecipitation achieves better NP quality with a smaller size and narrower distribution due to turbulence-based intensive mixing. Micro-fluidic nanoprecipitation allows precise control over the physicochemical properties of NPs but faces challenges such as system blocking and low productivity [118].

In the study mentioned above, dos Santos Alves et al. investigated starch nanoparticle production via nanoprecipitation, utilizing cassava and potato starches as carriers to stabilize phenolic compounds derived from green propolis extract [115]. The research also explored the antioxidant and antimicrobial activities of phenols stabilized with starch nanoparticles, along with their release characteristics under gastrointestinal conditions. The propolis extracts exhibited notable antioxidant and antibacterial properties, containing varying concentrations of p-coumaric acid, rutin, kaempferol, and quercetin. Starch nanoparticles demonstrated a bimodal distribution, with particle sizes below 340 nm. Moreover, the stabilization of phenols resulted in an increase in surface charge and hydrophobicity in the starch nanoparticles [115].

The investigation of Mehra and coworkers aimed to enhance the water solubility of berberine by fabricating berberine sodium alginate nanoparticles using a combined approach of a conventional nanoprecipitation method and ionic complexation [119]. The optimized nanoformulation exhibited a particle size ranging from 20 nm to 100 nm with an entrapment efficiency of 94.47%. The nanoformulation facilitated sustained release of berberine over approximately 48 h, following zero-order kinetics, and displayed enhanced in vitro antioxidant activity.

When fabricating polysaccharide nanoparticles (NPs), a significant consideration is **the crosslinking** of the polysaccharide to achieve stable particles [120].

Crosslinking profoundly influences the properties of polysaccharide NPs and their applications, including mechanical properties, swellability, and drug encapsulation/release [121]. Covalent crosslinking establishes irreversible connections, resulting in highly stable structures resistant to chemical stress such as solvent changes or a wide pH range. Common crosslinkers for polysaccharides include dialdehydes, which form connections via acetal formation, and multifunctional carboxylic acids, which create ester or amide bonds [122]. However, potential toxicity from remaining non-linked small molecule crosslinkers poses a concern for biomedical applications. Ionic crosslinking, another method, involves the addition of oppositely charged small ionic molecules like tripolyphosphate (TPP) or ions with multiple vacancies like Ca^2+^ [123]. Electrostatic interactions connect the crosslinker with the polysaccharide, resulting in crosslinking. While ionic crosslinking offers lower chemical and physical stability compared to covalent crosslinking, this defined instability can be advantageous for controlled degradation, particularly in drug delivery applications [122].

Nanocellulose antioxidant (Aox-NCC) was incorporated as a reinforcement and crosslinking agent in nitrile butadiene rubber (NBR) composites [124]. The crosslinking density and volume of rubber bonded in the matrix increased with the addition of Aox-NCC up to 3 phr. This study demonstrates that Aox-NCC effectively reinforces NBR composites, enhancing their mechanical properties and stability.

Furlani et al. recently discussed the formation and stability of complex coacervates based on chitosan and hyaluronan [125]. In the synthesis protocol, trace amounts of tripolyphosphate, a multivalent anion, act as a supplementary crosslinker for chitosan. Various types of hydrochloride chitosan with different average molar masses were chosen to create a diverse range of formulations. The study explores parameters such as coacervate size, surface charge, and uniformity in relation to chitosan characteristics. The stability of coacervates in phosphate-buffered saline was assessed using scattering techniques, including dynamic light scattering and small-angle X-ray scattering.

**Coacervation or polyelectrolyte complexation** is a relevant crosslinking method involving the use of oppositely charged polysaccharides and/or polyelectrolytes to form coacervate structures via electrostatic interactions [126]. The complex coacervation technique involves mixing aqueous solutions of two polymers with opposite charges, making them suitable for NP preparation due to its simplicity and mild conditions and eliminating the need for organic solvents. This method has been extensively employed to synthesize and enhance natural polysaccharide NPs by utilizing oppositely charged polyelectrolytes. When solutions containing oppositely charged polyelectrolytes (polycation and polyanion) are mixed, an equilibrium between the dense coacervate phase and the dilute solution phase is established. The coacervate, enriched with polyelectrolytes, forms as a result of electrostatic interactions and entropy gain during the process. A notable aspect of complex coacervation is its ability to modulate the net charge of the resulting NPs by adjusting the ratio of polyelectrolytes, leading to a core-shell structure with one component in excess at the outer shell. This approach is highly biocompatible when no toxic small molecule crosslinkers are used, relying on charge density and distribution along the chain for stability. Chitosan is commonly used as the cationic polysaccharide in polyelectrolyte complex formation, often combined with negatively charged polysaccharides like hyaluronic acid, dextran, and carboxymethyl cellulose [127]. pH, ionic strength, and temperature play crucial roles in the stability and preparation of coacervate particles, which can also be utilized in forming layer-by-layer assemblies [128].

In the study of Morais et al., bacterial nanocellulose membranes were loaded with ionic liquids based on phenolic acids. These ionic compounds, with improved solubility and bioavailability, were prepared by combining the cholinium cation with anions derived from caffeic, ellagic, and gallic acids. These membranes revealed a controlled ionic liquids dissolution rate in the wet state and high antioxidant activity. The work demonstrated the potential of nanostructured cellulose membranes loaded with phenolic-based ionic liquids for skin treatment [129].

The **self-assembly** of polysaccharide chains offers another supramolecular approach for nanoparticle formation [100]. Typically, hydrophilic polysaccharides are modified with hydrophobic components such as bile acids, fatty acids, or cholesterol, inducing aggregation in aqueous environments and resulting in micellar structures [130].

In this manner, nanoparticles with a hydrophobic core and a hydrophilic polysaccharide shell are produced. The core can act as a reservoir for hydrophobic drugs or other molecules. The properties of the micellar nanoparticles can be customized through the polysaccharide conjugate used, such as molecular weight or the ratio of hydrophobic to hydrophilic components, as well as through the preparation process, including the kinetics of micelle formation or the choice of solvent system. Breitenbach and colleagues took this approach a step further by synthesizing an amphiphilic block copolymer of dextran and hydrophobic acetylated dextran to form micellar nanoparticles [131]. Thus, a fully polysaccharide-based amphiphilic block copolymer was introduced. Additionally, a relevant structure akin to micelles, namely polysaccharide–drug conjugates, merits discussion. Instead of incorporating non-functional hydrophobic molecules to induce supramolecular aggregation, hydrophobic drugs can also be directly conjugated to the polysaccharide. This results in the formation of polysaccharide nanoparticles that serve as carriers for drug delivery. A crucial aspect is ensuring that the connection between the drug and polysaccharide is cleavable in the body, ideally at the desired site of action. Moreover, other methods of aggregation or crosslinking can potentially be combined with polysaccharide–drug conjugates [132].

For example, fucoidan, a sulfated polysaccharide, possesses significant antioxidant activity, yet its negative surface charges hinder oral absorption [133]. To address this, oligofucoidan self-assembled with an oppositely charged polysaccharide, chitosan, to form chitosan–fucoidan polysaccharide nanoparticles, sized 190–230 nm. These NPs were evaluated for radioprotective properties in mice exposed to 5 Gy radiation. NPs effectively prevented radiation-induced lipid peroxidation and restored intestinal antioxidant levels (*p* < 0.05). Histological analysis confirmed their radioprotective effect, preserving crypt and villi integrity in the small intestine. These NPs show promise as radioprotective agents against radiation-induced intestinal injury.

**Emulsion-based techniques**, considered a standard method for polymer NP preparation, are also highly versatile in synthesizing polysaccharide NPs [100,120,134]. Emulsification involves the creation of a metastable dispersion, typically of oil and water, known as an emulsion, which remains in equilibrium despite its tendency to separate into two phases. In general, emulsions are categorized based on the type of dispersed phase and dispersion medium. These categories include O/W direct emulsion and water-in-oil (W/O) inverse emulsion, where water or oil serves as the continuous phase, respectively. In contrast to the nanoprecipitation method, which is a simple one-step process, the preparation of organic nanoparticles like polysaccharide nanoparticles using the emulsification method involves a two-step procedure. Nanoparticles can be produced from the emulsion through diverse techniques, including solvent evaporation, solvent diffusion, and reverse salting-out methods. The process of creating polysaccharide nanoparticles through the emulsification method involves several steps. First, a solution containing polysaccharides is prepared by dissolving them in deionized water or another suitable solvent. Next, this solution is dispersed within an oil phase to create either O/W or W/O emulsions, achieved through stirring or ultrasonication. Finally, the polysaccharide nanoparticles are formed through either internal or external gelation. In external gelation, crosslinkers diffuse from an external source into the polysaccharide emulsion, while in internal gelation, the crosslinkers are already present within the droplets prior to gelation [135].

Antioxidant-loaded protein–polysaccharide nanoparticles are investigated in stabilizing and delivering curcumin with high internal phase Pickering emulsions. Resveratrol-loaded α-lactalbumin–chitosan particles were utilized to stabilize and deliver curcumin in high internal phase Pickering emulsions (HIPPEs) [136]. Confocal laser scanning microscopy demonstrated effective adsorption of resveratrol-loaded α-lactalbumin–chitosan nanoparticles on the oil/water interface, forming a gel-like structure around the oil droplets. HIPPEs displayed excellent stability, with 75.4% curcumin retention for HIPPEs with resveratrol-loaded α-lactalbumin–chitosan particles after 30 days. In comparison to medium-chain triglycerides, HIPPEs enhanced lipolysis and curcumin bioaccessibility, with lower lipolysis extent. The presence of resveratrol significantly restrained lipolysis, indicating HIPPEs as promising systems for delivering lipophilic curcumin.

Curcumin has also been the subject of research conducted by Zhang and colleagues. They conducted a study aimed at enhancing the dispersibility and antioxidant efficacy of curcumin. They successfully prepared curcumin emulsions coated with cellulose particles of various structures and examined the structural features, stability, and antioxidant properties of the emulsions [137]. DPPH scavenging activity of curcumin emulsion stabilized by cellulose-based nanoparticles significantly increased on increasing the hydrolysis duration, which was much higher than that of pure emulsion and curcumin/water.

**Figure 6:**
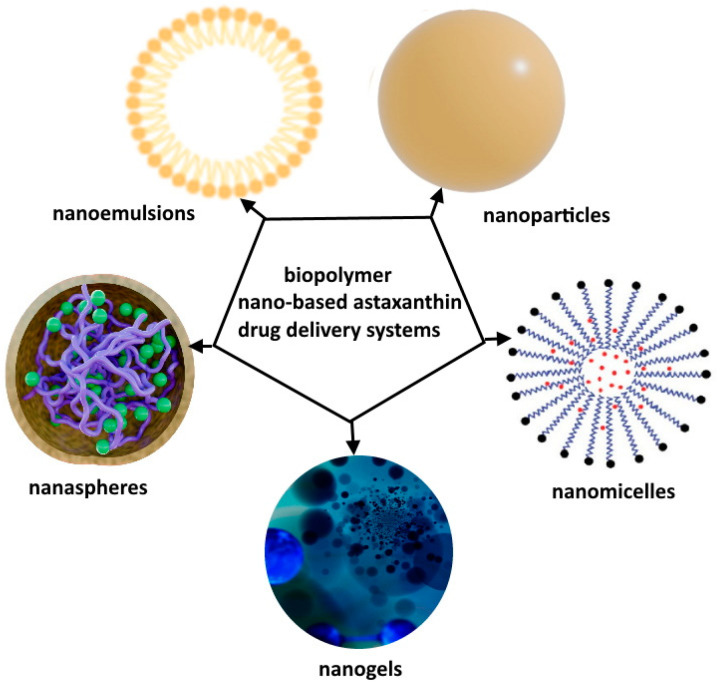
Different nanoforms of biopolymers with astaxanthin.

Astaxanthin has great potential in the prevention and treatment of clinically related diseases due to its antioxidant activity. So far, various nanoforms of particles with astaxanthin have been synthesized (Figure 6) [138,139]. Recently, Sun et al., in their review, provided some ideas on the research trends and applications of astaxanthin delivery systems [140]. They stated that joint use of two or more materials can significantly enhance the stability and that encapsulation systems slow down the degradation of astaxanthin. Studies and applications are mostly focused on (nano)emulsions and systems with biopolymers [140]. For example, Shanmugapriya et al. recently investigated the effects of low-level laser therapy with cellulose nanocrystal/cellulose nanofibril-loaded astaxanthin nanoemulsion for the induction of apoptosis via ROS-dependent mitochondrial dysfunction in cancer cells under photobiomodulation [141].

#### 2.2.2. Nanogels

Polysaccharide-based hydrogels at nanoscale, known as nanogels, can entrap drugs within their network structure. Nanogels offer high water content and flexibility, making them suitable for delivering both hydrophilic and hydrophobic drugs [142].

For example, alginate nanohydrogels have found application in antioxidant delivery, often employing multi-functional crosslinkers and combining them with other polymers to enhance mechanical properties and optimize bioactive release profiles [143].

Xu and colleagues were inspired by hyaluronic acid’s targeting ability toward CD44-overexpressed inflammatory cells, along with the redox regulation capacity of diselenide compounds. They developed an oral nanoformulation called diselenide-bridged hyaluronic acid nanogel to treat colitis by scavenging reactive oxygen species [144].

Sanchez et al. investigated the utilization of cellulose nanofibers and lignin in the production of ultra-light aerogels for biomedical purposes. They developed aerogels with varying lignin content (0–30 wt%) and different concentrations of the crosslinking agent Fe^3+^ (25–100 mM) [145].

#### 2.2.3. Nanospheres and Nanocapsules

**Figure 7:**
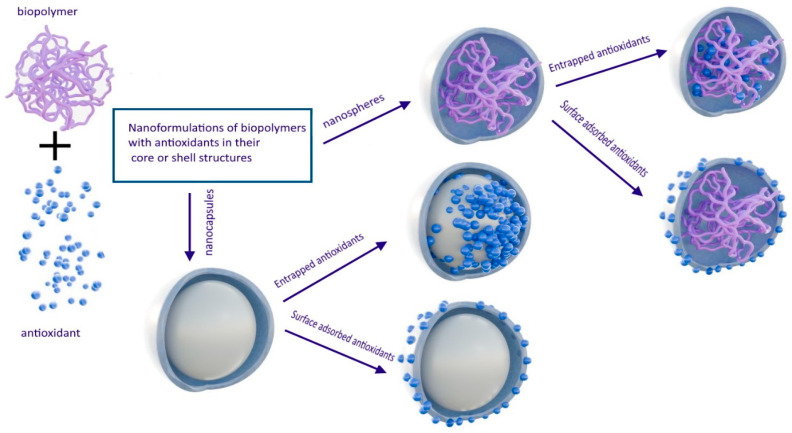
Schematic illustration of the designs of nanospheres and nanocapsules.

Polysaccharide nanospheres and nanocapsules are formed to encapsulate drugs in their core or shell structures, providing controlled release and protection to the loaded drugs (Figure 7). Recently, Sarma et al. achieved successful encapsulation of resveratrol within a nanosized core composed of chitosan, which was then coated with a pectin shell to create a drug delivery vehicle capable of prolonging the entrapment of resveratrol. The core-shell nanoparticles produced underwent various physicochemical characterization methods for evaluation. In vitro drug release analysis demonstrated the sustained release capability of the core–shell nanoparticles, extending the release of resveratrol for nearly 30 h [146].

Xu and coworkers applied core–shell hemp seed globulin alginate nanoparticles for Cannabisin A constructed by electrostatic interaction to encapsulate Cannabisin A [147]. The particles exhibited a spherical shape, with a Z-average diameter of 562.57 ± 8.59 nm, a ζ-potential value of −35 mV, and the highest LA value of 13.48 ± 0.04 μg mg^−1^.

#### 2.2.4. Nanoemulsions

Polysaccharides can be used to create stable nanoemulsions, which are colloidal dispersions of oil and water stabilized by surfactants or polymers. Nanoemulsions can encapsulate lipophilic drugs and improve their bioavailability. These polysaccharide-based nanoformulations offer several advantages in drug delivery, including targeted delivery, sustained release, reduced side effects, and improved therapeutic efficacy. Researchers continue to explore and optimize these formulations to overcome challenges such as stability, scalability, and fine-tuning drug release profiles for various applications in medicine. Modified starches are favored among biopolymer-based emulsifiers due to their robust stability against varying ionic strengths, pH levels, and high temperatures. Octenyl succinic anhydride-modified starches have emerged as effective emulsifiers and stabilizers in numerous studies, facilitating the encapsulation of lipophilic bioactive compounds like curcumin, flavoring oils, and resveratrol [148]. Ali et al. conducted a study to formulate nanoemulsions loaded with fat-soluble vitamins (β-carotene and α-tocopherol), employing starches and Tween 80 as stabilizers. They also explored the impact of co-entrapped antioxidants (cinnamaldehyde) and carrier oils. The nanoemulsions, containing fat-soluble vitamins and cinnamaldehyde emulsified with propylene glycol unsaturated (PGU) and Tween 80, exhibited exceptional physical stability during one month of storage at various temperatures [148].

### 2.3. Surface Modification

The majority of the methods and research outlined above typically incorporate a stabilizer or surfactant in their formulations. Surface modification using stabilizers in biopolymer nano-based drug delivery systems involves the attachment of stabilizing agents onto the surface of nanoparticles to enhance their stability and antioxidative properties [149].

These stabilizers can be natural or synthetic compounds that interact with the surface of nanoparticles to prevent aggregation, degradation, or undesired interactions with the surrounding environment. Common stabilizers include surfactants, polymers, proteins, and lipids, which form a protective layer around the nanoparticles, shielding them from external factors such as pH changes, temperature variations, and enzymatic degradation. This surface modification technique helps to improve the performance and efficacy of biopolymer nano-based drug delivery systems, making them more suitable for biomedical applications, including antioxidative therapy [150].

Examples of stabilizers commonly used for surface modification in biopolymer nano-based drug delivery systems include Tween, Span, sodium dodecyl sulfate (SDS), and bovine serum albumin (BSA), which form a monolayer or bilayer around the nanoparticles, reducing surface tension and preventing particle aggregation [151,152].

Polyethylene glycol (PEG) is a commonly used polymer stabilizer that can be conjugated to the surface of nanoparticles to improve their stability and biocompatibility [153].

Bovine serum albumin (BSA) and gelatin are protein stabilizers that can form a protective layer around nanoparticles, preventing their aggregation and enhancing their stability in biological fluids [154].

Phospholipids such as phosphatidylcholine and cholesterol can be used as stabilizers to form lipid bilayers or vesicles around nanoparticles, providing a biocompatible and stable coating [155]. These stabilizers can be incorporated into the formulation of biopolymer nano-based drug delivery systems during the preparation process to modify the surface properties of the nanoparticles and improve their stability, biocompatibility, and antioxidative properties.

### 2.4. Factors Influencing Degradation and Drug Release Kinetics

Several factors can influence the degradation and drug release kinetics of biopolymer nano-based drug delivery systems with antioxidative properties. These include the composition and properties of the biopolymer used, the presence of stabilizers or surfactants, the method of nanoparticle fabrication, the pH and temperature of the surrounding environment, and the chemical nature of the loaded drug. Additionally, interactions between the biopolymer matrix and the drug molecule, as well as any crosslinking agents or modifiers, can also impact degradation and release kinetics [156,157,158].

Different biopolymers have varying degrees of stability and degradation rates. For example, alginate and chitosan have different degradation profiles due to their chemical structures and susceptibility to enzymatic degradation [159]. The molecular weight, degree of crosslinking, and porosity of the biopolymer matrix can also influence degradation and drug release kinetics [160]. Stabilizers or surfactants are often added during nanoparticle fabrication to improve stability and prevent aggregation. These additives might alter the interactions between the biopolymer and the drug molecule, thereby influencing drug release kinetics [161]. The method used to fabricate biopolymer nanoparticles, such as nanoprecipitation, emulsification, or coacervation, has an impact on the size, morphology, and structure of the nanoparticles, which in turn can affect degradation and drug release behavior [162]. Environmental conditions, such as pH and temperature, exert an effect on the degradation of biopolymer nanoparticles. For example, changes in pH can trigger swelling or degradation of the biopolymer matrix, leading to accelerated drug release [163,164,165]. The chemical properties of the loaded drug, such as solubility, hydrophobicity, and stability, can influence its release from biopolymer nanoparticles. Hydrophobic drugs may be more effectively encapsulated within the hydrophobic core of nanoparticles, while hydrophilic drugs may diffuse more readily through the biopolymer matrix [166]. Crosslinking agents or modifiers used during nanoparticle fabrication can affect the stability and degradation of biopolymer nanoparticles. These interactions might alter the structure of the biopolymer matrix and impact drug release kinetics [162].

By considering these factors, researchers can optimize the design of biopolymer nano-based drug delivery systems to achieve controlled and sustained drug release with antioxidative properties, thereby enhancing their therapeutic efficacy.

## 3. Polynucleotide-Based Nanoformulations

Polynucleotide nanoparticles are a type of nanoparticles that are commonly used for the delivery of antioxidants. These nanoparticles are made up of polynucleotides, which are long chains of nucleotides (DNA or RNA) [167,168]. It should be noted here that gene-based nanoparticles such as DNA, mRNA, siRNA, and others can also be considered as a type of biopolymer-based nanosystem by themselves. Even though they differ from traditional biopolymers like polysaccharides or proteins in their composition and structure, they share common characteristics (function, biocompatibility, and tailorability) that make them suitable for drug delivery applications. Like other biopolymers, gene-based nanoparticles are generally biocompatible and biodegradable, minimizing the risk of adverse effects and promoting the safe and effective delivery of therapeutic agents. They are derived from natural biological materials, making them suitable for use in medical applications. Gene-based nanoparticles can be engineered to modulate their properties, such as size, surface charge, and targeting ligands, to optimize their performance for specific applications [169]. This flexibility allows researchers to tailor the nanoparticles for different therapeutic purposes and target diseases.

When antioxidants are loaded into polynucleotide nanoparticles, they can be protected from degradation and delivered to specific target cells or tissues in the body. This targeted delivery helps to enhance the antioxidant’s therapeutic effects while minimizing side effects. The use of polynucleotide nanoparticles for antioxidant delivery has shown promise in various biomedical applications, including the treatment of oxidative stress-related diseases and disorders. These nanoparticles can be engineered to release antioxidants in a controlled manner, providing sustained protection against oxidative damage. Overall, polynucleotide nanoparticles offer a promising approach for the delivery of antioxidants, with potential applications in the fields of drug delivery and healthcare. Some examples of polynucleotide nanoparticles used for the delivery of antioxidants include polymeric DNA nanoparticles, RNA-based nanoparticles [170], DNA origami nanoparticles [171], aptamer-functionalized nanoparticles [172], and lipid-conjugated DNA nanoparticles [173].

Polymeric DNA nanoparticles are constructed using DNA as the building blocks and can be loaded with antioxidants for targeted delivery [174,175].

The stationary DNA branched junctions render DNA an excellent building material for constructing DNA nanostructures using single-stranded sticky ends. This approach has led to the creation of various DNA nanostructures, including 3D DNA cubes, truncated octahedrons, and Borromean rings, by linking the branched DNA junctions [171,176,177,178].

DNA origami nanoparticles are created by folding DNA strands into specific nanostructures, allowing for precise loading and delivery of antioxidants [179,180]. Ma et al. employed DNA origami as a nanomedicine to target rheumatoid arthritis therapy by scavenging reactive oxygen species and nitric oxide [181]. Lipid modifications can be added to DNA nanoparticles to improve stability and enhance cellular uptake for antioxidant delivery [173]. RNA molecules can be engineered into nanoparticles for the delivery of antioxidants, providing a versatile platform for therapeutic applications [182]. Aptamers, which are short nucleic acid sequences, can be attached to nanoparticles to target specific cells or tissues for antioxidant delivery [183]. Aptamer nanoparticles used for the delivery of antioxidants include, for example, aptamer-conjugated liposomes, aptamer-functionalized gold nanoparticles, aptamer-coated silica nanoparticles, aptamer-conjugated dendrimers, etc. Liposomes are lipid-based nanoparticles that can be functionalized with aptamers to target specific cells or tissues for antioxidant delivery [184]. Polymeric nanoparticles can be modified with aptamers to enhance their targeting capabilities and improve the delivery of antioxidants to desired locations. Gold nanoparticles can be functionalized with aptamers to target specific receptors on cells and deliver antioxidants for therapeutic purposes [185]. Recently, bioconjugation of silica nanoparticles with aptamers was performed using EDAC and sulfo-NHS by Grechkinto et al. [186,187]. Dendrimers are highly branched nanoparticles that can be functionalized with aptamers to enhance their targeting and delivery of antioxidants to specific cells or tissues [188].

There is a diverse range of polynucleotide nanoparticles that can be utilized for the delivery of antioxidants in biomedical applications. Polynucleotide-functionalized nanoparticles offer a promising approach for precise and efficient antioxidant delivery in therapeutic interventions.

## 4. Protein-Based Nanoformulations

Proteins represent abundant, renewable, and cost-effective resources. Moreover, certain protein-based macromolecules, such as viruses, can self-assemble, forming hollow nanoarchitectures with precise geometries and highly organized capsids. Their empty internal cavities can serve as reservoirs to transport and deliver pharmaceuticals, diagnostics, and imaging agents [189]. The term ‘cage’ suggests that nanoparticles can be released in response to environmental stimuli in certain cases, capitalizing on unique chemical and physical disparities at the target site.

Protein nanoformulations offer several advantageous properties, positioning them as a material of choice in drug delivery and tissue engineering. The most significant advantages are (i) biocompatibility—proteins, being fundamental biomolecules in all living organisms, exhibit minimal toxicity, particularly when compared to synthetic polymers, and their ability to absorb water and generate space repulsion enhances nanoparticle stability and reduces recognition by the immune system; (ii) biodegradability—proteins undergo breakdown in the body, with the resulting amino acids utilized by surrounding tissues for protein synthesis or energy production; (iii) eco-friendly—proteins are abundant and renewable resources found in nature, sourced from plants, animals, humans, and other organisms; (iv) high drug binding capacity—proteins possess numerous functional groups, enabling significant drug binding through mechanisms such as electrostatic interactions, hydrophobic interactions, and covalent bonds; (v) targeting—proteins’ structural and sequential diversity, along with their abundant functional groups, facilitate drug binding to specific sites within the protein and attachment of various targeting ligands to the protein nanoparticle; and (vi) efficient internalization—protein nanoparticles are typically efficiently taken up by cells through various mechanisms.

Despite the numerous benefits of proteins, several processing-related limitations persist, spanning from the extraction process to the isolation of the final biomaterial. Given their completely natural origin, the ultimate properties of proteins are heavily influenced by the source of the primary starting material.

Thanks to their conformation and structure, proteins are recognized for their crosslinking capabilities and their ability to form hydrogels. Hydrogels represent the most abundant form of protein-based DDSs with a significant number of products in the market. Collagen and gelatin are widely utilized components in numerous cosmetic products and serve as starting materials in the development of hydrogels for the controlled release of various active substances. Recently, nanoparticles emerged as an ideal cargo in protein-based hydrogels. A few review papers can be found regarding this topic [190,191,192]. Due to their properties, protein-based hydrogels meet all criteria for wound dressing applications [193,194,195,196]. Protein nanoparticles represent innovative drug delivery technologies and systems in the pharmaceutical field. Several review papers have been published recently regarding this topic [42,197,198].

Various methods have been developed for the production of protein-based nanoparticles. Generally, these procedures can be classified into several groups, often combining elements from multiple methods. Some of these methods are already mentioned above, such as self-assembly, crosslinking, and coacervation, and they are also widely used in the case of proteins. Selected natural proteins/peptides can **self-assemble** into intricate structures via non-covalent interactions. Modulating pH or salt concentration allows controlled assembly and disassembly of protein particles. **Aggregation of denatured proteins** is based on the fact that protein denaturation exposes hydrophobic amino acids, prompting protein aggregation into dense particles. **Crosslinking** is typically necessary to stabilize these particles due to weak Van der Waals forces. Regarding the crosslinking of proteins in emulsions, protein nanoparticles can be formed through emulsion droplet creation. Proteins dissolved in an aqueous phase are mixed with an organic solvent, followed by crosslinking for stabilization before removing the solvent. Covalently modifying proteins with hydrophobic polymers (**assembly of protein–polymer conjugates**) induces self-aggregation into micellar nanoparticles. In aqueous environments, proteins predominantly surface, while hydrophobic polymers occupy the core. Mixing oppositely charged proteins and polymers can yield nanoparticles via strong electrostatic interactions, known as **coacervation**. Particle formation relies heavily on pH and ionic strength, ensuring homogeneous distribution inside. **The desolvation method** relies on the utilization of a desolvating (dehydrating) agent, such as ethanol or acetone. These agents induce changes in the polymer’s structure and decrease its solubility, leading to the precipitation of polymeric particles.

All these methods address a broad spectrum of proteins, each presenting unique advantages and drawbacks depending on the intended application [199]. For instance, although denatured protein aggregation is common in drug delivery, the loss of the protein’s native structure may restrict its usefulness outside of this context. Moreover, while these methods can enhance cellular uptake or circulation times, they may also influence immunogenicity. Uncontrolled protein aggregates or extensive denaturation might trigger immune responses, potentially leading to adverse effects like severe allergic reactions [200].

Although the focus of this review paper is to summarize results regarding the utilization of biopolymers in designing nano-based DDSs with antioxidative properties, it is worth noting that some of the protein-based molecules possess intrinsic antioxidative properties and as such could be used alone or in combination with other materials. Among them, the most prominent are certainly peptides, followed by poly(amino acids) and mimetic enzymes. These bioactive peptides, whether derived from dietary proteins or synthesized, have demonstrated substantial ROS scavenging activity, offering potential benefits for human health promotion. Review papers that address this topic have been published recently [201,202,203,204,205,206].

The one that particularly deals with peptide nanoformulations was reported by Bhargavi Ram Thimmiah et al. [207]. In these papers, readers can find diverse information regarding types, production, and molecular mechanisms of peptide antioxidative action and their potential applications.

Based on the literature data, all 20 essential amino acids present in the human body can be incorporated into the design of peptides and/or poly(amino)-based ROS scavengers. These ROS scavengers can be correlated with chronic degenerative diseases such as cardiovascular and cerebrovascular diseases, inflammatory-induced cancer, rheumatoid arthritis, and diabetes.

The primary production method for peptides involves enzymatic hydrolysis of both animal and plant proteins to generate protein hydrolyzates, which can be further refined through membrane ultrafiltration to obtain peptide fractions based on size and finally undergo separation via column chromatography to isolate pure peptides. While the structural mechanisms underlying enhanced antioxidant activity may vary, protein hydrolyzates and peptide fractions containing predominantly low-molecular-weight peptides have consistently demonstrated potent antioxidant properties. Moreover, protein hydrolyzates and peptides containing hydrophobic amino acids such as Valine (Val) or Leucine (Leu) in their N-terminal regions, as well as aromatic amino acids (Phenylalanine (Phe), Tryptophan (Trp), and Tyrosine (Tyr)), sulfur-containing amino acids (Cysteine (Cys) and Methionine (Met)), and the imidazole ring-containing Histidine (His), have been identified as possessing robust antioxidant properties.

### 4.1. Nanoparticle-Based Systems

The main limitation of peptides in achieving their full potential is low stability, especially considering oral administration. For instance, Casein phosphopeptide (CPP) exhibits promising potential in chelating transition metal ions to mitigate ROS generation. However, CPP is susceptible to hydrolysis and enzymatic degradation. To address this issue, Xiaoyan Ma and colleagues synthesized CPP nanoparticles via the covalent assembly of Genipin and CPP (GCPP NPs), aiming to enhance CPP stability in physiological environments and bolster its in vivo antioxidation capabilities for treating inflammatory bowel disease (IBD) [208]. The obtained particles were spherical with an average diameter of ~100 nm. As the authors demonstrated, GCPP NPs were able to resist the harsh pH/enzymatic conditions found in the oral administration route. In vitro assays confirmed the potent antioxidative properties of GCPP NPs, as assessed by ABTS and MTT assays. Furthermore, employing a DSS-induced mouse colitis model, specific accumulation and significant therapeutic efficacy of orally administered GCPP NPs were observed. Notably, GCPP NPs exhibited benign antioxidant activity, effectively scavenging ROS, and demonstrated passive accumulation at inflamed sites. Treatment with GCPP NPs led to a trend of rehabilitation in terms of body weight and colon length in DSS-induced colitis in mice.

Based on the ability of ferritin to form spherical nanocages with a hollow center for storing iron, Xiaoyu Xia and his colleagues used recombinant human H-ferritin nanocages loaded with natural antioxidative lycopene molecules (LYC) [209]. The main goal of this research was to enhance LYC permeability into the brain and investigate its regulatory mechanism on neurodegeneration. For this purpose, a mouse model of D-galactose-induced neurodegeneration was used, and the reported results demonstrated improved mouse behavior in a dose-dependent manner after treatment with this ferritin-based delivery system. Among other activities, it decreased neuronal damage and prevented excessive accumulation of neurotoxic proteins in the hippocampus of mice. Thus, the authors consider this system to be an excellent strategy for reducing oxidative damage in the brain and inhibiting the development of neurodegenerative diseases.

Berberine (BBR) is an isoquinoline alkaloid with diverse clinical therapeutic applications. Among them, the antioxidative aspect is one of the most prominent. However, as is case with many phytopharmaceuticals, its efficacy is compromised by low water solubility, absorption, and cellular bioavailability. Fatema A. Younis et al. address these limitations by encapsulating BBR within bovine serum albumin nanoparticles (BBR-BSA NPs) [210]. BBR-BSA NPs and BSA NPs were synthesized using the desolvation method, which involves drop-by-drop addition of ethanol at a rate of 1 mL/min to the BBR-BSA complex solution, followed by crosslinking using a specific volume of 8% glutaraldehyde. The hydrodynamic radius of the obtained particles was ~150 nm. Compared to BBR alone, the authors reported that BBR-BSA nanoparticles exhibit superior free radical scavenging, antioxidant, anti-hemolytic, anticoagulant, and antimicrobial properties in vitro. Furthermore, in a stressed pancreatic rat model induced by a high-fat, high-sucrose diet plus carbon tetrachloride injection, oral administration of BBR-BSA NPs significantly restored peripheral glucose metabolism and insulin sensitivity. Moreover, BBR-BSA NPs improved pancreatic β-cell homeostasis, upregulated pancreatic antioxidant mechanisms, suppressed oxidant generation, and mitigated oxidative injury in stressed pancreatic tissues.

A similar approach to improving β-Carotene (BC) bioavailability and efficiency in expressing antioxidant activity was performed by Hye Gyeong Yang et al. [211]. To overcome this limitation, BC was encapsulated within BSA nanoparticles via high-speed homogenization. Following homogenization, spherical BC-BSA-NPs were obtained with an average diameter of 112 nm and high encapsulation efficiency. BC encapsulated within the BSA-NPs remained stable throughout the preparation process and exhibited sustained release kinetics in PBS at 37 °C over 3 days. Evaluation with 2,2-diphenyl-1-picrylhydrazyl and ferric reducing antioxidant power assays confirmed the high antioxidant activity of BC-BSA-NPs.

According to the paper published by Yuting Fan and colleagues, pea protein isolate (PPI) nanoparticles have a good potential as a nano-carrier for resveratrol (RES) by providing an increased bioavailability, as well as improved antioxidant activities [212]. In this study, a cold-gelation approach utilizing calcium ions to induce protein crosslinking was employed for synthesizing PPI nanoparticles. This method eliminates the need for organic solvents and offers precise control over the size and zeta-potential of the nanoparticles. The thorough investigation of the diverse properties of the obtained system allowed the authors to determine the impact of pH and calcium concentration on the formation and characteristics of the PPI nanoparticles, the formation and stabilization mechanism of calcium-induced PPI nanoparticles, and the binding interaction between PPI nanoparticles and RES. As the authors emphasized, the novelty of this work lies in its pioneering approach to fabricating efficient plant-derived PPI nanoparticles via calcium-induced protein crosslinking specifically tailored for the stabilization and delivery of RES.

### 4.2. Hydrogel-Based (Nano)Formulations

A novel methacrylate anhydride-modified hyaluronic acid–collagen hybrid hydrogel with incorporated polypyrrole (PPy) nanoparticles was developed by Chengheng Wu et al. [213]. This hydrogel was used as a conducive 3D niche for encapsulated bone marrow-derived mesenchymal stem cells (BMSCs), offering neuroprotective and neuroinductive effects. The PPy nanoparticles expressed dual function: antioxidative activity to shield the BMSCs from oxidative damage, and electroconductivity, essential for intracellular electrical signal transmission and response to external electrical stimulation. The antioxidative activity of the hybrid hydrogels was characterized by the DPPH scavenging method and by the detection of ROS level and peroxidative damage in vivo. Following 7 days of implantation, the ROS level at the lesion site was assessed using dihydroethidium (DHE) staining. Remarkably, ROS levels exhibited a significant decrease compared to both the control and blank probe groups, attributed to the presence of the antioxidative PPy nanoparticles within the hydrogel. Additionally, the expression levels of 4-hydroxynonenal (lipid peroxidation product) and 8-hydroxy-2′-deoxyguanosine (marker of DNA oxidative stress) were substantially inhibited by this multifunctional hydrogel.

Interestingly, increased stability of sensitive enzymes such as Catalase (CAT) can be accomplished by simple immobilization within the hydrogel. CAT represents one of the key parts of an antioxidative shield in living organisms. According to Heidi Mohamed Abdel-Mageed et al. [214], a biocompatible hydrogel composed of gelatin (Gel) and alginate (Alg) (Gel–Alg) and prepared utilizing calcium chloride as an ionic crosslinker can be used to effectively inhibit the prolongation of the wound healing process mediated by ROS. Entrapment efficiency of 92% was achieved with a combination of 2% Gel and 1.5% Alg. The CAT immobilized within the Gel–Ag hydrogel exhibited enhanced stability over a wide pH range (4 to 9) and temperature range (20 to 60 °C) compared to free CAT. Furthermore, this hydrogel demonstrated remarkable reusability, retaining 52% of its original activity after 20 consecutive catalytic runs. Finally, in a thermal injury model, the authors reported significant differences in lesion sizes between the treated group and the control group observed 24 h post-injury. 

The protective function of hydrogels was also observed in the work conducted by Zhiwei Shen and his colleagues [171]. This study introduces a hydrogel, ECF-5, composed of a free radical scavenging and biodegradable supramolecular peptide, tailored as a 3D scaffold for cardiomyocyte cultures. Remarkably, this peptide hydrogel fosters cardiomyocyte migration and proliferation while shielding them from oxidative stress. Moreover, the hydrogel degrades as cells proliferate, potentially avoiding capillary thrombosis. The other examples of protein-based hydrogels with antioxidative activity are summarized in Table 2.

### 4.3. Other Formulations

Another study which highlights the potential of biopolymer-based carriers for enhancing the water solubility, chemical stability, and biological activities of hydrophobic polyphenols was performed by Jiang Yi et al. [226]. In their paper, pea protein isolate **nanofibrils** were fabricated and utilized as nanocarriers for encapsulating, stabilizing, and delivering resveratrol (RES). Following the initial dissolution–precipitation–dissolution process of the protein, fibrils were obtained through sequential heating and rapid cooling under reduced pH. These PPI nanofibrils exhibited significantly higher surface hydrophobicity compared to native PPI and had nanoscale dimensions with widths of 10 nm and average lengths of 1.0 μm. Fluorescence analyses revealed a high binding constant between PPI nanofibrils and RES. When compared to free RES, the aqueous solubility of RES was dramatically improved by approximately 1000-fold upon complexation with PPI nanofibrils. Furthermore, antioxidant capacity assays using DPPH and ABTS radicals demonstrated a pronounced enhancement in the antioxidant activity of RES upon complexation with PPI nanofibrils.

In the work performed by Jiabo Shi et al., an interesting approach was reported for fabrication of self-assembled, flexible, and antioxidative collagen **nanocomposite film** [227]. The authors used supramolecular interactions of type I collagen and tannic acid (TA)-functionalized 2D synthetic clay nanoplatelet Laponite (LAP) to create transparent collagen nanocomposite film with enhanced versatile mechanical properties, along with the 71% increase in antioxidant capability. With their straightforward fabrication process, the authors foresee that this approach opens extensive possibilities for the design and fabrication of bionanocomposites for various emerging applications.

## 5. Polyester-Based Nanoformulations

Although some parts of the research community define biopolymers strictly as those found in living organisms, the broader definition will include all materials that are typically composed of naturally occurring monomers and are often involved in biological processes. Here, DDSs based on aliphatic polyesters and polyhydroxyalkanoates will be also considered.

Among polyester DDS formulations, the most dominant nanoforms are **nanoparticle-based systems** (nanocapsules, nanospheres), **nanofibers**, and nanoporous systems (**membranes and hydrogels**). When it comes to nanoparticle-based DDSs, synthesis approaches can be generally divided into procedures from preformed polymers and direct polymerization of monomers. The latter is more appropriate for other types of synthetic polymers that cannot be classified as biopolymers. The most utilized techniques with preformed polymers are emulsification–solvent evaporation and diffusion, nanoprecipitation, the supercritical anti-solvent method, and salting-out. The choice of preparation method depends on several factors, starting from the physicochemical properties of polymers and active components, requirements of the final system (size, morphology), application requirements (active/passive targeting, capsule/matrix architecture, etc.), and many others. The discussion on the synthesis techniques for various DDS nanoformulations based on polyesters is a sufficient source for a separate review paper thanks to an enormous number of papers reported on this topic. However, their utilization for systems with antioxidative activity is still insufficient compared to other activities.

Recently, the microfluidic approach emerged as a most promising technique to overcome the limitations of conventional techniques such as reproducibility and uniformity. Speaking generally, microfluidics utilizes already-mentioned and well-known techniques (precipitation, emulsion–solvent evaporation) combined with the specific design of the reactors. This unique design provides strict control of the reaction conditions and parameters which in turn results in high uniformity and size control for the final system particles. Thanks to the technological advancements in the production of these reactors, particularly in the thin network of channels, and mixing zones in parallel chips, the scale-up of microfluidic production is drastically increased. A comprehensive review of papers regarding microfluidic-assisted production of PLGA and PLA DDSs can be found elsewhere [228,229]. In terms of the present topic, two review papers were published in the last five years analyzing the potential of microfluidics for the encapsulation of compounds with antioxidative properties [230,231]. In another research paper, the authors compared the rutin-loaded PLGA nanoparticles synthesized by microfluidic and conventional (bulk) approaches [232]. The parameters such as decreased size and increased encapsulation efficiency were in favor of microfluidics. Moreover, the microfluidic approach resulted in higher burst release and overall faster rutin release, measured in biorelevant media and PBS.

Aliphatic polyesters are a class of synthetic polymers that are derived from aliphatic (non-aromatic) monomers. These polymers are characterized by their linear, chain-like structure and are widely used in various applications due to their biodegradability, biocompatibility, and tunable properties. Thanks to the presence of ester linkages, they are susceptible to hydrolytic degradation. Polyesters include poly(lactide)s (PLAs), poly(caprolactone)s (PCLs), poly(glycolide)s (PGAs), poly(lactide-co-glycolide) (PLGAs), poly(dioxanone)s (PDOs), poly(butyrolactone)s (PBLs), and many others obtained by a combination of the aforementioned techniques. When it comes to DDSs, the most investigated and utilized ones are PLA, PLGA, and PCL [41]. Since the first two are obtained by polymerization of naturally occurring monomers, only those are discussed.

It can be said that PLGA represents the gold standard in the selection of synthetic polymers when designing drug delivery systems. The impact and significance of PLGA on this field of research can be easily verified through literature analysis. After searching the SCOPUS database with the following terms: PLGA and DRUG and DELIVERY and SYSTEMS, it can be found that 7510 papers have been published so far, of which 2798 were published in the period from 2019 to present (the search was within abstracts, titles, and keywords). More interestingly, when the search is translated to patents, it results in 49,540 matches. From this number, 17,492, or 35%, were reported in the past 5 years. In the context of this review, over the past five years, a significant number of papers have been published that incorporate PLGA in various formulations as drug delivery systems. Nevertheless, most of these papers deal with antioxidative properties tangentially. Some of the review papers that explain applications of PLGA-based DDSs have been published recently [233,234,235,236]. The results from papers focused on the antioxidative properties of PLGA-based DDSs are summarized in Table 3.

For drug delivery systems, interactions with active substances (drugs) are very important. Aliphatic polyesters are generally considered as an inert carrier. However, in a study published recently, the effect of the incorporation of polyphenols extracted from sage and sol–gel derived from PCL and PLGA was evaluated regarding the degradation behavior [237]. The authors have found that polyphenols accelerate the degradation of both polymers. They proposed that this phenomenon is related to several actions: wettability improvement, acid catalysis, plasticizing effect, and porosity formation.

Another aspect of selecting the biopolymer as a drug carrier is the administration route or interaction with the biological environment at the site of administration. For instance, in a study published recently, the authors compared two systems for the delivery of polyphenolic compounds contained in cherry extract [238]. The first one showed PLGA NPs to be less toxic than the ones obtained from chitosan derivatives. Conversely, due to the mucoadhesive properties, the latter system managed to protect human umbilical vein endothelial cells more efficiently from oxidative stress and maintained its activity compared to non-encapsulated cherry extract and cherry extract encapsulated within PLGA NPS.

PLA stands out as a versatile biopolymer. It is easily synthesized from abundant renewable resources. Due to its biodegradability and thermoplastic properties, it finds diverse healthcare applications ranging from tissue engineering and regenerative medicine to orthopedic, cardiac, and dental uses, drug carriers, and skin and tendon healing [239,240]. Its inherent properties make PLA ideal for rapid prototyping and efficient manufacturing in 3D-printed constructs. This capability enables the creation of patient-specific tissue engineering scaffolds and facilitates the swift production of medical equipment, including personal protective equipment (PPE) crucial for ensuring the safety of healthcare workers during the COVID-19 pandemic. When it comes to DDSs, PLA is mainly used in conditions where strong stability and/or certain mechanical characteristics are required, for example, in the form of films, fibers, networks, and then nanoparticles. Additionally, when antioxidative properties are needed, the focus of many reported studies lies in the development of films and nanoparticles. These studies commonly employ strategies involving the impregnation or immobilization of various phenolic compounds such as thymol, carvacrol, limonene, cinnamaldehyde, lignin, quercetin, curcumin, and silver nanoparticles [241,242,243,244,245,246,247,248,249]. The remaining results are summarized in Table 3.

**Table 3:** Summary of published research that utilizes PLGA, PLA, or PHAs in DDSs with antioxidative properties.

	Active Agent	Form of DDS	Reported Results/Remarks	Reference
Nanoparticles	tanshinone IIA	PLGA-b-PEG-OH NPs; size 92 nm	In vitro—reduction in SOD activity. In vivo (ischemic stroke pigs)—reduced cerebral swelling, lesion volume, and improved White Matter Integrity	[250]
	epigallocatechin-3-gallate (EGCG)	PEGylated PLGA NPs, diameter 167 nm	Sustained-release profile up to 8 days. In vivo (subarachnoid hemorrhage SAH models)—superior antioxidative activity compared with unloaded EGCG. Combined with nimodipine = suppressed oxidative stress, Ca^2+^ overloading, mitochondrial dysfunction, and autophagy after SAH.	[251]
	phytol	PLGA NPs stabilized with PVA; average size 177 nm	In vitro—reducing ROS and RNS levels by activating the antioxidative defense system (superoxide dismutase and catalase) and restoring glutathione-metabolizing enzyme systems. In vivo (scopolamine-induced memory dysfunction in Wistar rats)—enhanced biodistribution and sustained release profile of phytol in the brain and plasma.	[252]
	rutin and the (*S*)-*N*-(2-oxo-3-oxetanyl)biphenyl-4-carboxamide derivative	PLGA NPs stabilized with poloxamer 188; average size 200 nm	In vitro—the multidrug formulation exhibited a dose-dependent enhancement of protective effect against H_2_O_2_-induced oxidative stress when compared to nanosystems containing the active compounds individually.	[253]
	naringin	PLGA NPs stabilized with PVA; Diameter 137 nm	Burst release in the initial 24 h followed by sustained release lasting for 10 days. DNA-binding activity was maintained after nanoencapsulation	[254]
	caffeic acid—covalently bonded	PHB–diethanolamine (PHB-DEA) nanoparticles; Diameter 232 nm	In vitro—powerful antioxidant capacity and bacterial inhibition.	[255]
	quercetin	nano-silica and PLGA nanocomposite; NPs Size 100–200 nm	In vitro and in vivo testing—potential application for cardiovascular diseases.	[256]
Nanoporous formulations (membranes, films) hydrogels	niobium carbide nanosheets	Hydrogel: PLGA–PEG–PLGA triblock copolymer	In vitro and in vivo confirmation of ROS—scavenging activity/strategy for the treatment of diabetic wounds.	[257]
	AuNPs and antimicrobial peptides (Os)	Polydopamine-modified PLGA membrane	In vitro—bactericidal and antioxidant effects. In vivo (full-thickness skin defect model in rats)—combined with electrical stimulation—acceleration of vascularization, collagen deposition, and promotion of wound healing.	[258]
	oregano essential oil	PLGA/gelatin-based nanofibrous membrane with spinnable bioactive glass particles	In vitro—rapid hemostasis, improved chemotactic response, antibacterial and anti-inflammatory response. In vivo (rat tail amputation model, an ear artery injury model, and a liver trauma model in rabbits)—rapid hemostasis and significant tissue regeneration.	[259]
	curcumin	PHB–chitosan film	In vitro drug release—sustained pattern: 32% after 12 h, 48% after 20 h, and around 69% after 36 h.	[260]
	ammonium derivatives of tannic acid	network *mcl*-PHAs containing sulfonate groups	In vitro DPPH assay—high radical scavenging activity of 82%, stable for 5 months in a buffered physiological environment.	[261]
	ascorbic acid	P(3HB-co-3HV) copolymer	In vitro DPPH assay—increased scavenging effect.	[262]
Fibers/filaments	lignin	PLA fibers for 3D printing	In vitro DPPH assay—high radical scavenging activity of 80%. Meshes with curcumin for wound dressing.	[263]
	mango leaf extract	PLA filament for 3D printing	In vitro DPPH assay—reduction of 88%	[264]
	lignin	PLA fibers, electrospinning; Diameters 314–587 nm	In vitro, DDPH and ABTS—ultrafine fibers containing 2.5% lignin exhibited the highest antioxidant activity, with around 70%.	[265]

Polyhydroxyalkanoates (PHAs) represent a class of polyesters naturally synthesized by various microorganisms, often through bacterial fermentation of sugars or lipids. They are biopolymers naturally produced as an energy source by certain bacteria and organisms. When certain nutrients are limited, and carbon is abundant, bacterial cells synthesize PHA granules for long-term survival. Microorganisms capable of producing PHA include *Cupriavidus necator*, *Chromatium vinosum*, and *Pseudomonas aeruginosa*. Depending on the chain length, PHA can be classified into short-chain-length PHAs (monomers of three to five carbon atoms), medium-chain-length PHAs (monomers of six to fourteen carbon atoms), and a less common class, long-chain-length PHAs (with monomers exceeding fourteen carbon atoms). In the realm of nanomedicine, PHAs are highly favored due to their remarkable properties such as high loading capacity, biocompatibility, lack of toxicity, and biodegradability. Unlike other bioplastics, PHAs excel in medical applications because their monomers, such as 3-hydroxybutyric acid (3HB) and 4-hydroxybutyric acid (4HB), are recognized by the human body as degradation products, facilitating their bioresorption. Excellent review papers regarding the utilization of PHAs in drug delivery systems have been published recently [266,267,268,269].

Moreover, an interesting study was performed by Olga Cañadas and her colleagues, where they evaluated the potential application of PHAs as a drug delivery system for inhalation [270]. The authors’ findings suggest that PHA nanoparticles hold promise for pulmonary drug delivery due to the ability of the nanomaterial to translocate through the interfacial monolayer into the aqueous phase, facilitating the delivery of the cargo to its intended target site. Despite their advantageous properties, several characteristics of PHAs represent obstacles to their widespread application. These include high hydrophobicity, low thermal stability, and slow degradation rates. However, the most significant drawback is their high production costs. The high cost arises from the necessity for large quantities of high-purity substrates, as well as labor-intensive production and downstream processing. Polyhydroxybutyrate (PHB), a type of short-chain-length polyhydroxyalkanoate, stands out as the most extensively studied PHA polymer. It can be accumulated by both native and recombinant microorganisms, often reaching up to 80% of the cell’s dry weight. Due to the excellent biocompatibility and yet with processing issues, most of the research highlighting the utilization of this polymer is based on coatings applications, where diverse antioxidative and antimicrobial agents were immobilized or incorporated in PHB carriers [271,272,273]. Additionally, other examples of PHA utilization in DDSs with antioxidative properties are given in Table 3.

## 6. Looking Ahead

Biopolymer-based nano-drug delivery systems with antioxidative properties represent a promising avenue for innovation in drug delivery, offering potential benefits for both clinical and industrial translation. There are some emerging trends, challenges, and prospects related to this topic. For example, researchers are focusing on developing personalized drug delivery systems using biopolymer-based nanoparticles to enhance the efficacy of treatment and reduce side effects [274]. By tailoring drug delivery to individual patients, clinicians can optimize therapeutic outcomes and improve patient compliance.

Also, there is a growing trend towards combining antioxidants with drugs in nano-formulations to improve therapeutic outcomes and minimize oxidative stress-related damage. In the context of clinical translation, selecting a pair of biopolymer antioxidant agents imposes the primary determinant of the efficacy of the system’s application. For example, when zein and PLGA were compared in the form of nanoparticles as the carriers of rutin, the system with zein demonstrated several advantages such as (i) release profile—prolonged release of rutin caused by higher loading capacity, (ii) higher in vitro antioxidant activity—indicating a synergy between the inherent antioxidant properties of the protein and the pharmacological effects of the active compound, and (iii) pronounced intracellular localization [275]. The progress in DDS production, potentially resolving similar needs in the future, lies in artificial intelligence (AI). The integration of AI into DDS production is swiftly evolving, promising to reduce both the time and costs associated with developing new DDS while also enhancing ecological considerations for demanding synthesis. Consequently, it will streamline the path to clinical trials and eventual commercialization.

Advances in nanotechnology are enabling the design of targeted drug delivery systems that can specifically deliver drugs to diseased tissues or cells, enhancing treatment effectiveness. In 2023, the nano-based drug delivery market in the U.S. exceeded USD 63.55 billion and is anticipated to reach approximately USD 140.27 billion by 2033, with a compound annual growth rate (CAGR) of 8.23% from 2024 to 2033 [276]. The escalating prevalence of chronic diseases like diabetes, cancer, and cardiovascular conditions has increased the demand for innovative medications featuring targeted delivery for various chronic diseases. All of them require antioxidative effects as a part of symptomatic therapy. For instance, diabetes prevalence globally is on a sharp rise. As per the *International Diabetes Federation (IDF) Diabetes Atlas Tenth Edition 2021*, around 537 million adults aged 20–79 were diagnosed with diabetes in 2021. The same report projects a surge to 643 million by 2030 and 783 million by 2045. This mounting diabetes burden is expected to fuel demand for increasingly sophisticated drug delivery solutions for both treatment and diagnosis [277].

One of the challenges that remains on the commercialization route is regarding biocompatibility. Ensuring the biocompatibility of biopolymer-based nano-drug delivery systems is a major challenge, as some polymers may induce immune responses or toxicity. Also, maintaining the stability of the nanoparticles during storage and transportation is crucial for their effectiveness in drug delivery. The challenge is scalability. Scaling up the production of biopolymer-based nano-drug delivery systems for commercial applications while maintaining quality and consistency is a significant challenge [278].

In the context of industrial translation, ensuring the stability of nanoparticles during storage and transportation is paramount for their successful application in drug delivery on a large scale. Generally, nanoparticles are highly susceptible to aggregation, degradation, or changes in physicochemical properties when subjected to various environmental conditions such as temperature fluctuations, exposure to light, or mechanical stress during transportation and storage. Any alteration in nanoparticle stability can compromise their effectiveness in drug delivery, leading to inconsistent therapeutic outcomes. To address this challenge, rigorous quality control measures must be implemented throughout the manufacturing process to monitor and maintain nanoparticle stability. This includes optimizing formulation parameters, such as the choice of biopolymer, antioxidative substances, solvent, and stabilizing agents, to enhance nanoparticle stability and prevent aggregation or degradation. Additionally, appropriate packaging materials and storage conditions must be selected to minimize environmental exposure and preserve nanoparticle integrity during transportation and storage.

However, even with optimized stability measures, scalability remains a significant challenge in the industrial translation of biopolymer-based nano-drug delivery systems with antioxidative properties. Scaling up production from laboratory-scale to commercial-scale quantities while maintaining quality, consistency, and cost-effectiveness is a complex process that requires careful optimization of manufacturing processes, equipment, and raw materials. For instance, the transition from batch production methods typically used in research settings to continuous manufacturing processes required for large-scale production necessitates the development of robust and reproducible manufacturing protocols. Furthermore, scalability challenges may arise from differences in equipment capabilities, manufacturing conditions, and material properties between laboratory and industrial settings. To overcome scalability challenges, interdisciplinary collaboration between scientists, engineers, and industry professionals is essential to optimize manufacturing processes, address technical limitations, and ensure regulatory compliance. Additionally, investment in advanced manufacturing technologies, process automation, and quality assurance systems can help streamline production processes and enhance scalability. Overall, maintaining nanoparticle stability and achieving scalability are critical considerations in the industrial translation of biopolymer-based nano-drug delivery systems.

As already mentioned, biopolymer-based nano-drug delivery systems with antioxidative properties have the potential to enhance the efficacy of drug therapies by protecting drugs from degradation and targeting specific sites of action. By delivering drugs in a targeted manner, these systems can reduce systemic exposure and minimize the side effects associated with conventional drug delivery methods. There is also a prospect for developing theranostic systems that combine drug delivery with diagnostic capabilities, allowing for real-time monitoring of treatment effectiveness.

Overall, the development of biopolymer-based nano-drug delivery systems with antioxidative properties holds great promise for improving drug delivery efficiency and therapeutic outcomes. Researchers continue to explore novel approaches to address the challenges and leverage the opportunities in this exciting field.

## 7. Conclusions

In conclusion, the field of biopolymer-based nano-drug delivery systems with antioxidative properties is rapidly evolving, offering significant potential for enhancing drug delivery efficacy and therapeutic outcomes. Despite facing challenges such as biocompatibility, stability, and scalability, researchers are actively working to overcome these obstacles.

The emerging trends in customized drug delivery, combination therapies, and targeted delivery underscore the innovative approaches being pursued in this area. The prospects for improved drug efficacy, reduced side effects, and theranostic applications highlight the promising future of biopolymer-based nano-drug delivery systems.

As research in this field continues to advance, collaboration among scientists, engineers, and healthcare professionals will be crucial for translating these advancements into practical applications that benefit patients. Overall, the development of biopolymer-based nano-drug delivery systems with antioxidative properties represents a promising avenue for revolutionizing drug delivery and improving patient care.

## Figures and Tables

**Table 1:** The chemical structures of the reviewed biopolymers.

Polysaccharides	Chitosan	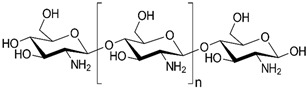
	Starch	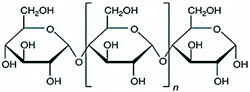
	Alginates	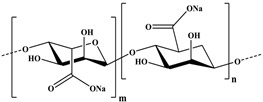
	Cellulose	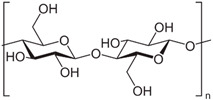
	Hyaluronic acid	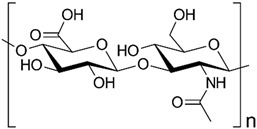
Polynucleotides	DNA	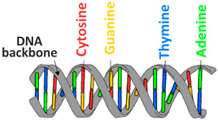
	RNA	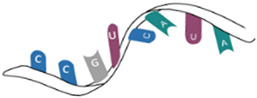
Proteins	Collagen	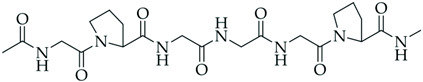
	BSA	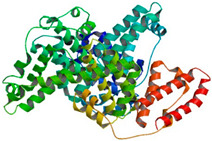
	Silk	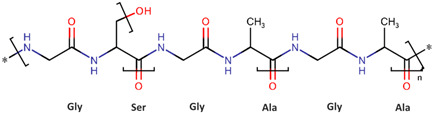
	Zein	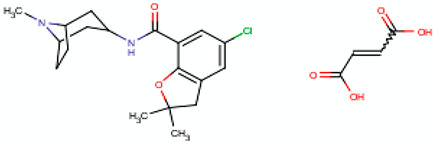
Polyesters	PLGA	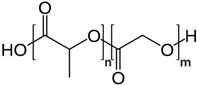
	PLA	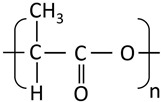
	Polyhydroxy-alkanoates	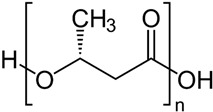

**Table 2:** Summary of published papers regarding protein-based hydrogels with antioxidative properties.

Active Agent	Composition of Hydrogel	Reported Results/Remarks	Reference
*Cannabis sativa* oil extract and AgNPs	Collagen	At 150 µL of *Cannabis sativa* oil extract high scavenger activity of 80%.	[215]
Caffeic acid (CA)	Collagen–chitosan	CA increases the dimensional stability of hydrogel after immersing in medium. Gradual release profiles within 8 h. DPPH·, ABTH+, and FRAP assays confirmed good antioxidative potential.	[216]
Caffeic acid (CA), ferulic acid (FA), and gallic acid (GA)	Collagen	Polyphenolic compounds induce higher swelling rate and enzymatic stability of hydrogel compared to pure collagen. Radical scavenging activity in the range 85–91%, highest for FA. In vivo tests—only hydrogels with FA generate an inflammatory process.	[217]
Vit. C	Electrospinning—zein + pectin	In vitro—DCFH-DA assay on HaCaT cells confirmed ROS decrease of 50%. In vivo test on a skin UVB-burn mouse model—significant reduction of inflammatory cytokines expression.	[218]
Carotenoids	Chitosan + protein isolate from blue crab	Low pH promotes release. In vitro, DPPH assay—radical scavenging 93–100%. In vivo rat model, acceleration of wound healing and complete healing.	[219]
Curcumin NPs	Gelatin–genipin	In vitro—hydrogel exhibits slightly higher antioxidative activity. Stability of curcumin NPs increased by 174%.	[220]
β-Carotene	Gelatin/polyglyceryl stearate/graphene oxide (GO)	Increasing GO concentration showed sustained release of β-Carotene. GPGO-3 β hydrogel showed the highest antioxidant potency (57.75%).	[221]
Au nanoparticles with lysozyme nanofibrils AuNPs@LNFs	Gelatin–hyaluronic Acid	Improved rheological properties, mechanical resilience, antioxidant activity, and electrical conductivity. The swelling and bioresorbable ratios were favorable at lower pH.	[222]
Thymol NPs	Chitosan–gelatin composite, gallic acid as crosslinker.	Composition of chitosan–type A gelatin with ratio 1:4 exhibits the best properties. Antioxidant capacity evaluated by DPPH, ABTS—radical inhibition 87% and 88.5%. FRAP assay—1085 µM Trolox equivalents.	[223]
Barbatimão extracts	Silk fibroin	Extractions in propylene glycol were superior to ethanol and result in better physical–chemical and structural performance of hydrogel. High antioxidant activity (ORAC and FRAP).	[224]
metal–organic framework nanozymes (CuTA@SF)	silk-based	In vitro—CuTA@SF hydrogel accelerates cell proliferation, enhances cell viability, and antioxidant and antibacterial properties. In vivo (rabbit model)—successful in situ osteochondral defect regeneration.	[225]
